# Isometric and isokinetic strength of lower-limb muscles in female athletes during different phases of menstrual cycle: a causal-comparative study

**DOI:** 10.1186/s12905-023-02819-w

**Published:** 2023-12-08

**Authors:** Fatemeh Pournasiri, Mostafa Zarei, Elena Mainer-Pardos, Hadi Nobari

**Affiliations:** 1 Department of sport Rehabilitation and Health, Faculty of sport science and Health, Shahid Beheshti University, Tehran, Iran; 2 University San Jorge, Autov A23 km 299, Villanueva de Gállego, Zaragoza, 50830 Spain; 3 Faculty of Sport Sciences, University of Extremadura, Cáceres, 10003 Spain; 4 Department of Exercise Physiology, Faculty of Educational Sciences and Psychology, University of Mohaghegh Ardabili, Ardabil, 56199-11367 Iran

**Keywords:** Female resistance, Anterior cruciate ligament, Injury, Menstruation, Strogen

## Abstract

**Background:**

Muscle strength is affected by different stages of the menstrual cycle in women. Since the strength of the lower limb muscles plays a significant role in the occurrence of anterior cruciate ligament injury, it seems necessary to study the strength of the lower limb muscles at different stages of this cycle to take preventive measures. Therefore, this study aimed to compare the isometric and isokinetic strength of lower limb muscles in female athletes at different menstrual cycle stages.

**Methods:**

The present study is of a causal-comparative type. Thirty-seven female athletes in anterior cruciate ligament injury high-risk disciplines participated in this study. Isometric and isokinetic knee extensors and flexors muscle strength status, including the traditional hamstrings/quadriceps (H/Q) ratios, were recorded at different stages of the menstrual cycle (follicular, ovulatory, luteal) by Biodex isokinetic dynamometry system 4. Then, the obtained results were analyzed by repeated measure analysis of variance.

**Results:**

Analysis of variance with repeated measures showed isokinetic and isometric strength in the knee flexor and extensor muscles at an angular velocity of 60˚/s in the ovulatory phase are higher than the follicular and luteal ones. The strength of the muscles declined during the follicular and luteal phases (*p* ≤ 0.05).

**Conclusions:**

Due to the negative effect of the follicular and luteal stages of the menstrual cycle on the strength of the flexor and extensor muscles of the knee, the risk of anterior cruciate ligament injury may increase during this period. Therefore, it is recommended that all female coaches and athletes take preventive measures during this period.

**Supplementary Information:**

The online version contains supplementary material available at 10.1186/s12905-023-02819-w.

## Introduction

One of the most common traumatic injuries to the knee ligament is the anterior cruciate ligament (ACL), often occurring in female athletes [1]. They are 2–10 times more at risk of ACL injury than males in sports involving deceleration, landing, and frequent rotations [1]. This injury rate among female basketball and soccer players has been reported at 2.8 and 2.3 cases per 10,000 individuals, respectively [2, 3]. In addition, a female who practices these team sports is the most vulnerable to a non-contact ACL injury [4, 5]. At least two-thirds of ACL injuries occur when an athlete performs acceleration, deceleration, jump-landing, pivoting, and cutting manoeuvres [3].

Additionally, treating ACL injury is expensive, and athletes with simultaneous injury to the meniscus or other ligaments in their knee develop early-onset osteoarthritis [6]. The physical, mental, and emotional damages of the ACL injury and its financial costs have prompted researchers to identify its modifiable risk factors. Previous studies have introduced neuromuscular risk factors as one of the risk factors for ACL injury [7–9]. Men and women have different neuromuscular patterns in the activation of lower-limb muscles when external traumatic forces enter [3]. In this regard, the role of muscle strength and hamstring: quadriceps muscle strength ratio as an important parameter in controlling knee joint movements has attracted the attention of researchers [10]. Most isokinetic evaluations have been conducted on the knee joint. Assessing the strength and function of and their balance/imbalance is considered one of the methods for preventing and treating knee injuries [11–13]. Muscle weakness and imbalance between the power and torque function of knee joint muscles, especially the hamstring and quadriceps, are among the leading causes of ACL injuries [14].

Along with various movements in the hip and knee joints, the hamstring stabilizes the knee joint and helps the ACL to prevent anterior tibial dislocation [15]. Further, the quadriceps muscle plays a crucial role in knee joint function. This muscle is effective in all knee movement and stability functions, as well as absorbing the forces on the joint [16], which cooperates with the posterior cruciate ligament (PCL) in maintaining the anterior-posterior stability of the knee. Given the importance of the functional balance of the muscles, some researchers have defined a certain ratio, called the hamstring to quadriceps muscle strength ratio (H:Q ratio) [10], which reduces stress on the knee’s ACL and helps to minimize injury to the posterior thigh muscles [17]. This ratio can be calculated by dividing the numerical values of the maximum concentric torque in the muscles through an isokinetic test, the standard level of which was reported between 0.5 and 0.8 [18]. A closer ratio to one lead to a lower possibility of knee injury [13].

Due to the physiological properties of the female body, the isokinetic strength of knee agonist and antagonist muscles for the movements of this joint is more important than the males [10]. As already mentioned, neuromuscular functions in the activation of lower-limb muscles differ between the sexes [6]. This issue can be attributed to hormonal factors and the type and amount of sex hormones in the female body. It seems that the secretion of sex hormones (estrogen and progesterone) from the body during the menstrual cycle, which occurs each month, plays a significant role in muscle strength and injury rate, the level of which affects the neuromuscular system directly and indirectly [19].

Previous studies have evaluated the parameters of women’s physical function during the different menstrual cycles. The research results among healthy female athletes with various hormone concentrations represented the non-uniform distribution of ACL injury during a menstrual cycle [20]. In addition, the injury prevalence is different at the various amounts of estrogen and progesterone [20]. This issue is ascribed to the direct effect of estrogen hormone on the collagen of ligament structure and its role in ligamentous laxity [21]. Regarding muscle strength, there is a hypothesis that the enhanced or rising estrogen level is associated with more muscle strength. Sarwar et al. found higher maximum handgrip strength during the first half of the menstrual cycle compared to the second [22]. Romero et al. reported a significant improvement in the strength of different body muscles by elevating estrogen value during the menstrual cycle [22]. Now, research is conflicting, with no consensus on whether muscle strength is affected by the menstrual cycle phase [23]. Accordingly, the results related to the strength variations following the change in the menstrual cycle phases need to be more consistent.

Furthermore, to our knowledge, no study has focused on isometric and isokinetic hamstring and quadriceps strength among female athletes in different phases of the menstrual cycle. This population forms an integral part of the sports community in Iran, among whom the risk of ACL injury is high. Based on the issue mentioned above and the adverse consequences caused by this severe injury, it is essential to examine hamstring and quadriceps strength as crucial variables to predict the incidence of ACL injury in the different phases of the menstrual cycle. Thus, the present study aims to compare the isometric and isokinetic strength of lower-limb muscles in female athletes during the various phases of the menstrual cycle. We have hypothesized that there is a difference between the isometric and isokinetic strength of the knee joint muscles of female athletes in different phases of the menstrual cycle.

## Methodology

### Participants

The study’s statistical population included female athlete students in Tehran, Iran. Statistical software (G*Power software vs. 3.1) was used to calculate the sample size. Given the study repeated measured ANOVA (1 group and three repeated measures), a medium overall effect size f = 0.25, an α-error = 0.05, and a desired power (1-ß error) = 0.8, and finally, the results revealed that actual power was 80.1% with a sample size of 37 participants. A total of 37 female athletes participated in the study. Table 1 summarizes the individuals’ demographic characteristics. The subjects were selected based on the purposive convenience sampling method among students with a three-year continuous activity in one of the high-risk sports for ACL injury (futsal, volleyball, basketball, handball, and martial arts). The subjects trained at least three sessions per week. The inclusion criteria were female athletes aged 18–25 years, undergraduate students, and a maximum menstrual cycle duration of 30 days.

Further, the other criteria can be mentioned as having no injury in the lower limb, not suffering from premenstrual syndrome, and not consuming contraceptives, dietary supplements and steroid drugs. The exclusion criteria included experiencing premenstrual syndrome due to nervous pressure or disease, taking hormonal medicine, lower-limb injury during the study, having irregular periods in last 6 months (Periods that occur fewer than 21 days or more than 35 days apart or Missing three or more periods in a row) [24], and inappropriate mental conditions to perform strength tests [21, 25]. Before the study began, all subjects were notified of the potential risks and benefits of participating in the research. All subjects signed an informed consent form to participate in the project. Before the start of the study, the study was approved by the ethics committee of the Shahid Beheshti University with the approval number IR.SBU.12/1,400,312. The study followed the Helsinki Declaration recommendations of Human Ethics in Research.

**Table 1:** Demographic characteristics of the subject (mean ± standard deviation)

Factor	Ag e(year)	(cm)Height	(kg)Body mass	(kg/m^2^) BMI
Mean±SD	21.65±3.5	171.14±9.2	64.07±4.9	23.63±2.2

### Study design

The present descriptive causal-comparative study was based on a single-group design. In this regard, the isometric and isokinetic strength of all subjects’ knee joint flexor and extensor muscles was measured in three stages during the different phases of the menstrual cycle on an isokinetic system at an angular velocity of 60˚/s on dominant leg (i.e., the leg the student preferably kicks the ball with) [26].

### Procedures

The statistical populations of the present study were undergraduate female athletes students of Shahid Beheshti University. After selecting the eligible athletes among the statistical population, they were invited to attend the pathology laboratory at the Faculty of Sport Science and Health of Shahid Beheshti University at the appointed time. The individuals who participated in the laboratory were given full explanations of how to conduct tests. They were first subjected to anthropometric measurements.

**Fig. 1:**
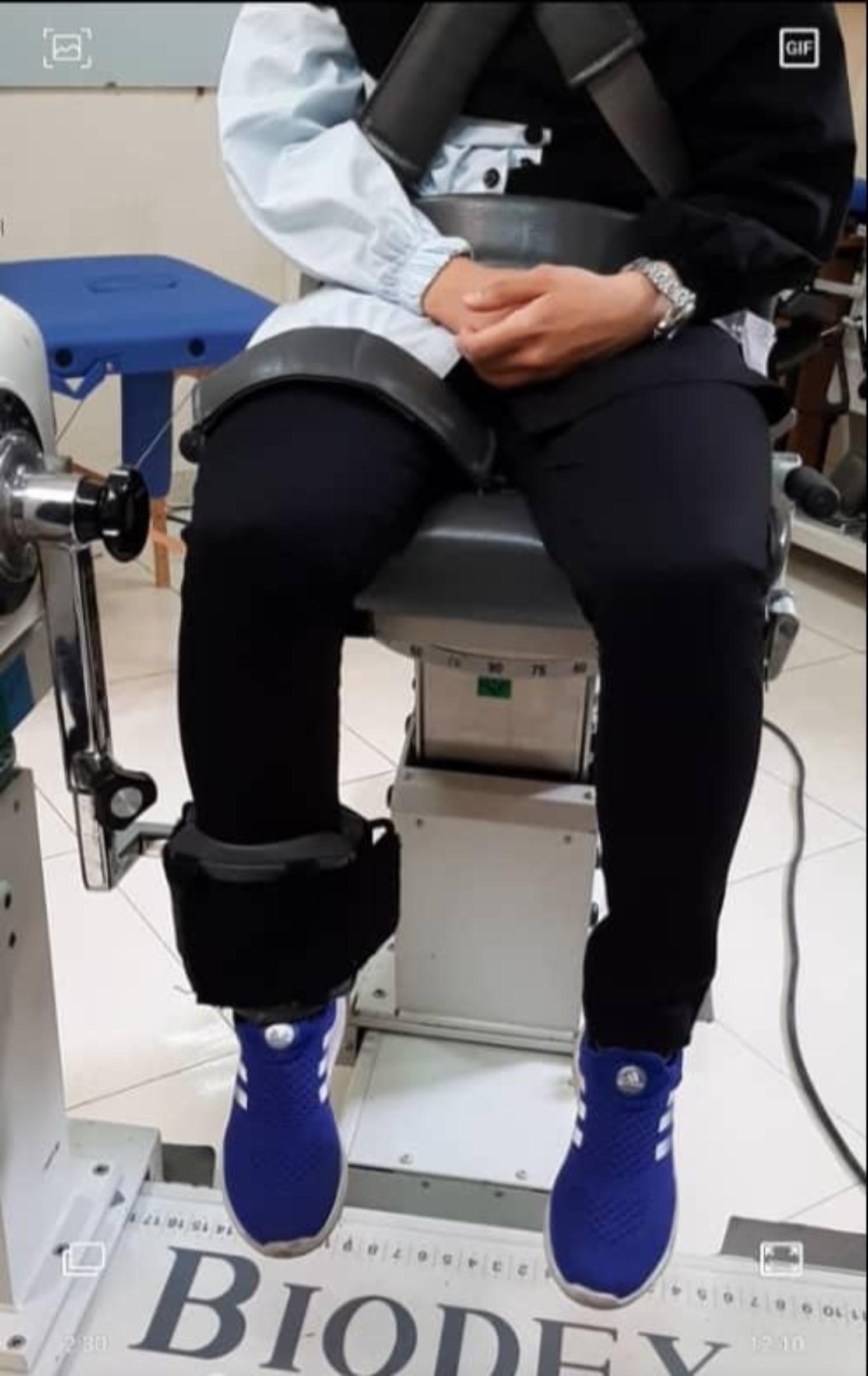
Measurement of knee flexor and extensor muscle strength by using the isokinetic instrument

The data were collected during a one-month process according to the menstrual cycle of the subjects. Each student attended the laboratory three times at the follicular (1–9 days), ovulatory (10–14 days), and luteal (15–28 days) phases [21]. It is worth noting that the exact timing of the phases was obtained by interviewing the individuals and their self-reporting about their cycle phases. Isokinetic dynamometers Biodex System 4 Pro (Biodex Medical System Inc, Shirley, NY, USA) is a reliable instrument with high intra-class correlation (ICC) [27] and was utilized to assess the isometric and isokinetic strength of the flexor and extensor muscles of the dominant leg, as well as the ratio of the two strengths at the angular velocity of 60˚/s [11]. The students performed a 15-minute warm-up on a cycle ergometer (Monarch Model 894E, Sweden) with a self-determined cadence (between 80 and 100 rpm) with the workload set to 75 W at the beginning of test sessions [28–30]. Afterwards, participants were asked to sit on the device’s seat to perform the isokinetic tests (Fig. 1). The seat back was regulated at 70–85˚, and the rotation axis of the device arm was precisely placed in front of the center of the lateral epicondyle of the dominant-leg thigh. After fixing the trunk and thigh to the seat, the upper part of the lateral malleolus was connected to the system’s rotation axis using unique cushions. The range of motion was 0–90˚, and the test velocity was 60˚/s [11]. The procedure mentioned above was repeated in the same way in the three measurements, followed by comparing the mean data of the three phases. Additionally, the evaluations of all three phases were conducted at a fixed time (10–12 a.m.) to minimize the adverse effect of daily activities on the results [25].

Further, before starting the test, each subject warmed up with an isokinetic device. Specific warm-up, including of three sub maximal contractions, and then three involuntary concentric contractions of maximum effort, was conducted to get the subjects prepared for the primary test. To ascertain knee strength for quadriceps and hamstring muscles, students performed five concentric contractions at 60°per second in a row. After two minutes of rest, subject performed five consecutive eccentric contractions at 60°per second. The average of the five strength trials was normalized to body mass, and the peak torque value was used for data analysis. Finally, any trial that resulted in a coefficient of variance (CV) greater than 15% was repeated after at least 2 min of rest. The functional ratio of isokinetic muscle strength was calculated by dividing the hamstrings’ eccentric peak torque by the quadriceps muscle’s concentric peak torque [31].

Regarding the isometric strength, the dynamometer was fixed at an angle of 60˚ based on the device manual. The isometric strength of the hamstring and quadriceps muscles was examined by performing three five-second contractions with a 20-second rest interval. The muscle strength was expressed in Nm according to the maximum generated torque [32].

### Statistical analyses

The Leven test determined the homogeneity of variables in the research groups, and the normality of data were evaluated using the Shapiro–Wilk test. Then, inferential tests were executed. Changes between the three-time points were assessed using a repeated-measures analysis of variance (ANOVA), followed by the Bonferroni post hoc test for pairwise comparisons. Data analysis was performed using SPSS software (23.0; IBM SPSS Inc., Chicago, IL, USA), and the significance level was set at *p* < 0.05 at all stages.

## Results

This study confirmed the assumption of data distribution normality using the Shapiro-Wilk test (*p* > 0.05). Therefore, the data related to the intended variables were normally distributed, revealing the establishment of the condition for applying parametric tests. The results of the repeated measure ANOVA of the parameters under study indicated a significant difference among the three phases (*p* ≤ 0.05), while the phases were not significantly different in terms of the H:Q ratio (*p* ≥ 0.05, Tables 2 and 3).

**Table 2:** Results of the repeated measure ANOVA of the isometric and isokinetic variables (absolute) in the three phases of menstrual cycle?0.05.Thank you so much."?>

Factor	Follicular	Ovulatory	Luteal	F	*p*
Isokinetic peak torque of knee extensors muscles at a speed of 60 (Nm)	115.4±18.8	131.8±21.1	116.6±9.69	12.0	*0.001
Isokinetic peak torque of knee flexor muscles at a speed of 60 (Nm)	85.2±6.81	98.7±8.22	86.7±8.14	11.17	*0.001
Isometric strength of knee extensor (Nm)	52.7±8.93	65.1±9.58	51.8±8.37	9.59	*0.001
Isometric strength of knee flexor (Nm)	67.1±6.29	82.7±10.8	69.1±9.32	14.6	*0.001
Flexor to extensor isokinetic power ratio	0.73±0.18	0.74±0.31	0.74±0.24	2.82	0.738

* Indicates significance at level *p* > 0.05

**Table 3:** Isometric and *isokinetic normalized peak torque (Nm/kg) during knee flexion and extension motion at different* phases of menstrual cycle

Factor	Follicular	Ovulatory	Luteal	F	*p*
Isokinetic peak torque of knee extensors muscles at a speed of 60 (Nm/kg)	1.81±0.42	2.03±0.61	1.81±0.54	10.51	*0.001
Isokinetic peak torque of knee flexor muscles at a speed of 60 (Nm/kg)	1.34±0.82	1.53±0.36	1.32±0.64	10.98	*0.001
Isometric strength of knee extensor (Nm/kg)	0.8±0.12	1.01±0.16	0.79±0.17	8.59	*0.001
Isometric strength of knee flexor (Nm/kg)	1.04±0.29	1.28±0.39	1.09±0.22	12.45	*0.001

* Indicates significance at level *p* > 0.05

Due to a significant difference among the three phases concerning the isokinetic and isometric strength of the knee flexor and extensor muscles, the Bonferroni post-hoc test was utilized for the paired evaluation of the changes between the phases. Based on the Bonferroni post-hoc test results, no significant difference was observed in the isokinetic and isometric strengths in the follicular and luteal phases (*p* > 0.05). However, the isokinetic and isometric strengths during the ovulatory phase differed significantly from those in the others (*p* ≤ 0.05, Table 4).

**Table 4:** Results of the Bonferroni post-hoc test of the isometric and isokinetic variables in the three phases of menstrual cycle

Factor	Phase	*p*	Confidence interval
Extensor isokinetic strength	Follicular-Luteal	0.621	-31.3-25.8
	Ovulatory-follicular	0.001*	-17.1 + 4.91
	Ovulatory-luteal	0.001*	-15.3 + 24.3
Flexor isokinetic strength	Follicular-luteal	0.153	-7.16-1.49
	Ovulatory-follicular	0.001*	-5.72 + 7.48
	Ovulatory-luteal	0.001*	-2.89 + 3.66
Extensor Isometric strength	Follicular-luteal	0.449	-11.1-4.62
	Ovulatory-follicular	0.001*	-2.51 + 8.36
	Ovulatory-luteal	0.001*	-14.2 + 3.97
Flexor isometric strength	Follicular-luteal	0.551	-23.1-13.56
	Ovulatory-follicular	0.001*	-9.15 + 3.71
	Ovulatory-luteal	0.001*	-16.3 + 5.17

* Indicates significance at level *p* > 0.05

## Discussion

This research aimed to compare the isokinetic and isometric strength of the knee joint muscles of female athletes in different phases of the menstrual cycle. The results of the present study represented a higher isokinetic and isometric strength in the knee flexor and extensor muscles at an angular velocity of 60 ˚/s in the ovulatory phase compared to the follicular and luteal ones. The strength of the muscles declined during the follicular and luteal phases. Therefore, the research findings confirm the research hypothesis.

Based on the results of the previous studies, the elevated or increasing concentration of estrogen is associated with greater muscle endurance and strength. For postmenopausal women who experience a decline in estrogen levels, estrogen replacement hormone therapy has been shown to effectively compensate for this decline. The results of our study also confirmed a significant difference among the three phases of the menstrual cycle (follicular, luteal, and ovulatory) (*p* ≤ 0.05). Gordan et al. reported significant changes in the maximum isokinetic torque of the knee extensor muscles during the menstrual cycle [33]. According to Sarwar et al., handgrip strength is higher during the first half of the menstrual cycle than in the second [22], which confirms the results of Philips et al. [34]. Sarwar et al. found that the quadriceps isometric strength of 10 untrained subjects enhanced by 11% in the mid-menstrual cycle (12–18 days or the ovulatory phase) [22]. These findings align with our results, suggesting that variations in muscle strength are indeed influenced by menstrual cycle phases.

The strength variations are seemingly consistent with estrogen and progesterone levels. Our study, in agreement with a recent meta-analysis that revealed that effects on muscle strength during different menstrual cycle phases are not significantly different [25], found that menstrual cycle phases do not significantly differ in terms of the hamstring-to-quadriceps (H:Q) ratio (*p* ≥ 0.05). Many women are more robust in the follicular phase, especially during ovulation when the estrogen-progesterone ratio is high. In the luteal stage, the strength immediately declines following the reversal of the estrogen-progesterone ratio. Thus, women gain muscle strength if strength training for injury prevention is performed when the strength is greater (i.e., the follicular phase). It seems that the more remarkable changes in muscle volume under better anabolic conditions contribute to the difference in muscle strength during the various phases of the menstrual cycle. This issue is confirmed by a higher rise in the muscle diameter in the ovulatory phase compared to the two others. The results of the previous studies have demonstrated the value of effective hormones on muscle strength improvement (e.g., testosterone and estradiol) peaks around the ovulatory phase. Therefore, these hormones are one of the factors for higher muscle strength compared to the other phases [35].

The present study showed differences in the isokinetic and isometric strengths of knee flexor and extensor muscles across the menstrual cycle phases. The Bonferroni post-hoc test indicated no significant difference in these strengths between the follicular and luteal phases (*p* > 0.05), but a significant difference during the ovulatory phase compared to the others (*p* ≤ 0.05). The fluctuation of sex hormones during the various phases of the menstrual cycle may influence neuromuscular and biomechanical characteristics. Estrogen and progesterone may affect the neuromuscular function of connective tissues if the fluctuations in the concentrations of the hormones change motor behavior [36]. The neuromuscular function of the tissues surrounding the ACL is vital for its survival. Furthermore, the quadriceps and hamstring muscles work together to provide functional stability to the knee. Knee weakness and alteration often cause ACL injury in muscle activation patterns [19]. Based on the literature review, steroid hormones may affect the neuromuscular function of connective tissues through CNS [36]. Nevertheless, these variations in muscle strength within the menstrual cycle may have implications for neuromuscular function and injury risk.

Previous studies have shown that some muscles’ timing and activation differ during the menstrual cycle [22, 37]. The semitendinosus muscle exhibits a significantly delayed activity during the luteal phase compared to the follicular one [19]. Additionally, a relationship is detected between a decline in joint stiffness and rotational movement control with the time delay in the onset of muscle activity [19]. Thus, a decrease in the hamstring muscle strength and H:Q ratio is an intrinsic risk factor for acute injury to knee joint ligaments [38]. Myer et al. reported that female athletes with ACL injuries have more hamstring muscle strength than men, although no difference was observed between the two sexes in quadriceps muscle strength [31]. According to our results, these findings highlight how menstrual cycle-related muscle strength fluctuations can impact muscle activation patterns and joint stability, particularly in the context of ACL injury risk.

Further, the individuals experiencing a reduction in the strength of the hamstring and quadriceps muscles were at risk of ACL injury, and an increase in the hamstring muscle strength could lead to less injury [39]. Our study’s results reinforce, the importance of the isokinetic strength of the knee flexor and extensor muscles can be ascribed to the crucial role of the hamstring and quadriceps muscles in controlling the players’ movements during reducing and increasing velocity, as well as orientation alteration, rotations, and knee joint movement control [11]. Many researchers have considered the H:Q ratio a critical factor for injury prevention since the player cannot control joint movement during eccentric activity if any of the muscles have less strength, leading to a higher risk of injury [10, 11, 14].

## Limitations

There are different methods to determine the menstrual phases. The self-report method was used in this study. Studies have shown that the self-reporting method is less accurate to determine the menstrual phases compared to other methods such as urinary LH testing and sex hormone measurement via saliva sample. The accuracy of self-report method is low compared to other methods, but due to its many advantages, this method has been widely used in studies, for example, Allen et al. in a review study have showed that the most common method of determining menstrual phases in different studies was self-reporting [40]. However, in generalizing and using the results of this study, attention should be paid to the method of determining the menstrual phases. Finally, in future studies, it will be essential to acquire hormonal data to definitively attribute the observed changes to the different phases of the menstrual cycle.

## Conclusion

Our findings indicate fluctuations in knee flexor and extensor muscle strength during the follicular and luteal phases. These fluctuations may have implications for the risk of ACL injury during these phases of the menstrual cycle among female athletes. Hence, female athletes and their coaches should focus on preventive measures during training and competitive periods.

### Electronic supplementary material

Below is the link to the electronic supplementary material.

**Supplementary Material 1**: The STROBE checklist is included in the supplementary files

## Data Availability

The data presented in this study is available from F.P. and M.Z.

## Abbreviations

ACL: Anterior Cruciate Ligament
H: Q ratio:Hamstring:Quadriceps ratio
PCL: Posterior Cruciate Ligament
SPSS: Statistical Package for Social Sciences
