# Supplementary material for: Isometric and isokinetic strength of lower-limb muscles in female athletes during different phases of menstrual cycle: a causal-comparative study
Source: BMC Womens Health. 2023 Dec 8;23:657. doi: 10.1186/s12905-023-02819-w (PMC10704707; doi:10.1186/s12905-023-02819-w)
Supplement: Supplementary file 1 — Supplementary Material 1: The STROBE checklist is included in the supplementary files [file 12905_2023_2819_MOESM1_ESM.pdf]

STROBE Statement—checklist of items that should be included in reports of observational studies

	Item No.	Recommendation	Page No.	Relevant text from manuscript
Title and abstract	1	(a) Indicate the study's design with a commonly used term in the title or the abstract	1	Line 2
		(b) Provide in the abstract an informative and balanced summary of what was done and what was found	1	Abstract
Introduction				
Background/rationale	2	Explain the scientific background and rationale for the investigation being reported	2-3	Introduction: paragraphs 1, 2,3,4
Objectives	3	State specific objectives, including any prespecified hypotheses	4	Introduction: last paragraph
Methods				
Study design	4	Present key elements of study design early in the paper	5	Study design Section
Setting	5	Describe the setting, locations, and relevant dates, including periods of recruitment, exposure, follow-up, and data collection	5	Procedures Section paragraph 1
Participants	6	(a) Cohort study—Give the eligibility criteria, and the sources and methods of selection of participants. Describe methods of follow-up<br>Case-control study—Give the eligibility criteria, and the sources and methods of case ascertainment and control selection. Give the rationale for the choice of cases and controls<br>Cross-sectional study—Give the eligibility criteria, and the sources and methods of selection of participants	4	Participants section paragraph 1
		(b) Cohort study—For matched studies, give matching criteria and number of exposed and unexposed<br>Case-control study—For matched studies, give matching criteria and the number of controls per case		Not applicable
Variables	7	Clearly define all outcomes, exposures, predictors, potential confounders, and effect modifiers. Give diagnostic criteria, if applicable	5-6	Procedures Section paragraphs 2,3,4
Data sources/ measurement	8*	For each variable of interest, give sources of data and details of methods of assessment (measurement). Describe comparability of assessment methods if there is more than one group	5-6	Procedures Section paragraphs 2,3,4
Bias	9	Describe any efforts to address potential sources of bias	6	Procedures Section Paragraph 4 line 177-178

Study size	10	Explain how the study size was arrived at	4	Participants section paragraph 1line 112-116

Continued on next page

Quantitative variables	11	Explain how quantitative variables were handled in the analyses. If applicable, describe which groupings were chosen and why	6	First paragraph Line 175-176
Statistical methods	12	(a) Describe all statistical methods, including those used to control for confounding	6	Statistical analyses Section
		(b) Describe any methods used to examine subgroups and interactions	6	Statistical analyses Section
		(c) Explain how missing data were addressed		We didn't have missing data
		(d) Cohort study—If applicable, explain how loss to follow-up was addressed<br>Case-control study—If applicable, explain how matching of cases and controls was addressed<br>Cross-sectional study—If applicable, describe analytical methods taking account of sampling strategy		Not applicable
		(e) Describe any sensitivity analyses		Not applicable
Results				
Participants	13*	(a) Report numbers of individuals at each stage of study—eg numbers potentially eligible, examined for eligibility, confirmed eligible, included in the study, completing follow-up, and analysed	4	Participants section paragraph 1
		(b) Give reasons for non-participation at each stage		Not applicable
		(c) Consider use of a flow diagram		Not applicable
Descriptive data	14*	(a) Give characteristics of study participants (eg demographic, clinical, social) and information on exposures and potential confounders	4	Table 1
		(b) Indicate number of participants with missing data for each variable of interest		Not applicable
		(c) Cohort study—Summarise follow-up time (eg, average and total amount)		Not applicable
Outcome data	15*	Cohort study—Report numbers of outcome events or summary measures over time		Not applicable
		Case-control study—Report numbers in each exposure category, or summary measures of exposure		Not applicable
		Cross-sectional study—Report numbers of outcome events or summary measures	7-8	Result section
Main results	16	(a) Give unadjusted estimates and, if applicable, confounder-adjusted estimates and their precision (eg, 95% confidence interval). Make clear which confounders were adjusted for and why they were included	7-8	
		(b) Report category boundaries when continuous variables were categorized	7-8	Tables 2,3,4
		(c) If relevant, consider translating estimates of relative risk into absolute risk for a meaningful time period		Not applicable

Continued on next page

Other analyses	17	Report other analyses done—eg analyses of subgroups and interactions, and sensitivity analyses	8	Table 4
Discussion				
Key results	18	Summarise key results with reference to study objectives	9	Discussion paragraph 1
Limitations	19	Discuss limitations of the study, taking into account sources of potential bias or imprecision. Discuss both direction and magnitude of any potential bias	11	Limitation section
Interpretation	20	Give a cautious overall interpretation of results considering objectives, limitations, multiplicity of analyses, results from similar studies, and other relevant evidence	9-11	Discussion section
Generalisability	21	Discuss the generalisability (external validity) of the study results	11	Paragraph 2
Other information				
Funding	22	Give the source of funding and the role of the funders for the present study and, if applicable, for the original study on which the present article is based	12	Funding section

*Give information separately for cases and controls in case-control studies and, if applicable, for exposed and unexposed groups in cohort and cross-sectional studies.

Note: An Explanation and Elaboration article discusses each checklist item and gives methodological background and published examples of transparent reporting. The STROBE checklist is best used in conjunction with this article (freely available on the Web sites of PLoS Medicine at http://www.plosmedicine.org/, Annals of Internal Medicine at http://www.annals.org/, and Epidemiology at http://www.epidem.com/). Information on the STROBE Initiative is available at www.strobe-statement.org.
